# Oligodendrocyte lineage is severely affected in human alcohol-exposed foetuses

**DOI:** 10.1186/s40478-022-01378-9

**Published:** 2022-05-14

**Authors:** Florent Marguet, Mélanie Brosolo, Gaëlle Friocourt, Fanny Sauvestre, Pascale Marcorelles, Céline Lesueur, Stéphane Marret, Bruno J. Gonzalez, Annie Laquerrière

**Affiliations:** 1 Department of Pathology, Normandy Centre for Genomic and Personalized Medicine, Laboratoire d’Anatomie Pathologique, Pavillon Jacques Delarue, CHU, Normandie Univ, UNIROUEN, INSERM U1245 and Rouen University Hospital, 1 Rue de Germont, 76031 Rouen Cedex, France; 2 UNIROUEN, INSERM U1245 F76000, Normandy Centre for Genomic and Personalized Medicine, Normandie Univ, Rouen, France; 3 Inserm UMR1078, Université de Bretagne Occidentale, Faculté de Médecine et Des Sciences de la Santé; Etablissement Français du Sang (EFS) Bretagne; Laboratoire de Génétique Moléculaire, CHRU Brest, Hôpital Morvan, Brest, France; 4 Department of Pathology, Bordeaux University Hospital, Bordeaux, France; 5 Pathology Laboratory, Pole Pathologie-Biologie, Centre Hospitalier Universitaire Brest, Brest, France; 6 Laboratory of Neurosciences of Brest, Faculté de Médecine et des Sciences de la Santé, Brest University, Brest, France; 7 Department of Neonatal Paediatrics and Intensive Care, Normandy Centre for Genomic and Personalized Medicine, Normandie Univ, UNIROUEN, INSERM U1245 and Rouen University Hospital, 76000 Rouen, France

**Keywords:** Oligodendrocyte precursors, PDGFR-α, Olig2, Myelination defects, Human foetal brain, Foetal alcohol syndrome, Immunohistochemistry

## Abstract

Prenatal alcohol exposure is a major cause of neurobehavioral disabilities. MRI studies in humans have shown that alcohol is associated with white matter microstructural anomalies but these studies focused on myelin abnormalities only after birth. Only one of these studies evaluated oligodendrocyte lineage, but only for a short period during human foetal life. As data are lacking in humans and alcohol is known to impair oligodendrocyte differentiation in rodents, the present study aimed to compare by immunohistochemistry the oligodendrocyte precursor cells expressing PDGFR-α and immature premyelinating/mature oligodendrocytes expressing Olig2 in the ganglionic eminences and the frontal cortex of 14 human foetuses exposed to alcohol from 15 to 37 weeks’ gestation with age-matched controls. The human brains used in this study were obtained at the time of foetal autopsies for medical termination of pregnancy, in utero or post-natal early death. Before birth, PDGFR-α expression was strongly increased in the ganglionic eminences and the cortex of all foetuses exposed to alcohol except at the earliest stage. No massive generation of Olig2 immunoreactive cells was identified in the ganglionic eminences until the end of pregnancy and the density of Olig2-positive cells within the cortex was consistently lower in foetuses exposed to alcohol than in controls. These antenatal data from humans provides further evidence of major oligodendrocyte lineage impairment at specific and key stages of brain development upon prenatal alcohol exposure including defective or delayed generation and maturation of oligodendrocyte precursors.

**Supplementary Information:**

The online version contains supplementary material available at 10.1186/s40478-022-01378-9.

## Introduction

Prenatal alcohol exposure (PAE) is a major non genetic cause of central nervous system (CNS) abnormalities resulting in irreversible life-long consequences which associate mental retardation or more specific neurocognitive disorders and behavioural disabilities [43]. Foetal Alcohol Syndrome (FAS) is a part of foetal alcohol spectrum disorders (FASD) which represents its most severe form. The diagnosis of FAS is based on three characteristics including in utero growth retardation (IUGR), craniofacial dysmorphism and CNS dysfunction. Children with FASD often suffer from motor delays, deficits in attention, learning and memory as well as in executive functioning and language with other less overt consequences of PAE that make up the constellation of CNS adverse outcomes. Nevertheless, children with FASD do not present the characteristic craniofacial dysmorphism and growth retardation [41].

In the mature brain, heavy chronic or binge drinking is responsible for a variety of brain injuries, notably a disproportionate loss of cerebral white matter which accounts for white matter atrophy, as glial cells are major targets of alcohol [48]. Alcohol has not only teratogenic properties but also devastating neurotoxic effects on the developing brain. Over the last decade, imaging studies with diffusion tensor imaging (DTI) have revealed changes in the organization and microstructure of callosal white matter with diffusion abnormalities extending beyond the corpus callosum in rodents and in humans with FASD, suggesting that several specific white matter regions, especially commissural, cingular and temporal connections along with deep grey matter areas are sensitive to PAE [25, 26, 31, 32, 34]. Microstructural abnormalities assessed by fractional anisotropy occur throughout the corpus callosum and have been correlated with impaired cognition in children with prenatal alcohol exposure [49]. A few years ago, it has been suggested in one human MRI study that PAE is associated with reduced white matter microstructural integrity from the neonatal period [10]. Whereas all these studies focused on newborns, children and adolescents with FASD, no human antenatal data concerning the generation of oligodendrocytes (OLs) in FAS or FASD have been reported so far, except for one human study concerning cases restricted to a short foetal period (12.2–21.4 weeks of gestation) [9]. As imaging studies indicate widespread white matter fibre tract anomalies in children and adults with FASD, it has been emphasized that PAE likely impacts on the programming of oligodendrocyte precursor cells (OPCs) [18, 48]. In the CNS, myelin is formed by processes emanating from OLs which are engaged or are preparing to engage in myelination [7, 39]. Myelination is one of the final stages of brain development, and apart from the brainstem and cerebellum which are progressively myelinated from the second half of pregnancy, this process takes place mainly during the first 30 years of postnatal life in humans [4, 25, 50].

OLs originate from neural stem cell-derived OPCs which express platelet-derived growth factor receptor-α (PDGFR-α), then differentiate into immature premyelinating OLs (pre-OLs) expressing oligodendrocyte lineage factor 4 and eventually into mature OLs expressing myelin oligodendrocyte glycoprotein, phospholipid protein and myelin basic protein (MBP) which contact axons and begin to produce myelin [7, 13]. Proliferating neural stem cells commit to the OL lineage under the influence of the transcription factors Olig1, Olig2, Nkx2.2, and Sox10 [13, 47]. In vitro studies have also revealed a primary role of Sonic Hedgehog (Shh) signalling by promoting expansion and specification of pluripotent progenitors into Olig2-positive late OPCs and immature OLs [35].

In rodents, the first wave of OPCs originates in the anterior entopeduncular area followed by two other waves arising from the lateral and caudal ganglionic eminences (LGE and CGE) [24]. OPCs require vessels as a physical substrate for migration and human OPCs have been shown to emerge from progenitor domains and to associate with the ab-luminal endothelial surface of blood vessels via Wnt-Cxcr4 (chemokine receptor 4) allowing them to “crawl along and jump between vessels” [45].

PDGF signalling is essential for the control of OPCs’ proliferation and differentiation [5]. These mitotic progenitors/precursors express a characteristic set of markers including PDGFR-ɑ. The first PDGFR-α expressing precursors have been identified in the forebrain of human foetuses 10 weeks of gestation (WG) onwards, and are predominantly produced in higher numbers in the ganglionic eminences (GE) around 15 WG [22]. In monkeys and humans, OPCs are produced in the outer subventricular zone just when the upper layer neurons are generated, allowing for a rapid expansion and folding of the cortical surface [37]. PDGFR-ɑ is rapidly downregulated when OPCs differentiate into non-proliferating OLs, contrary to oligodendrocyte lineage transcription factor 2 (Olig2) which is expressed in OPCs, pre-OLs and OLs, knowing that the increase in OL number during development depends on OPC proliferative capacities [11, 42]. OL lineage progression in cerebral white matter occurs similarly in humans and in rodents and is mostly composed of pre-OLs around 18–27 weeks of gestation in humans and of increasingly abundant MBP-positive OLs in full-term infants [3].

Human antenatal data concerning the generation and differentiation of OLs upon PAE are lacking even though alcohol is known to impair OL differentiation in other species. Therefore, we hypothesised that during the foetal period alcohol prevents the differentiation of PDGFR-α+ OPCs into Olig2+ pre-OL or mature OL, which could explain, at least partially, the myelination defects observed on imaging studies. To test this hypothesis, we compared using immunohistochemical techniques PDGFR-α and Olig2 expression in the GE and cortical plate (CP) of foetuses antenatally exposed to alcohol with age-matched controls. The objective was to confirm the pre-existing results in humans and to provide information regarding oligodendroglial lineage development over an extended neurodevelopmental period.

## Patients and methods

### Patients

As previously reported [28], the brains used in this study belong to the collection which has been declared to the French Ministry of Health (collection number DC-2015-2468, cession number AC-2015-2467, located in A. Laquerrière’s Pathology Laboratory, Rouen University hospital). In each case, the parents gave their informed written consent for neuropathological studies. Autopsies were carried out in accordance with our local ethic committee and the French law.

Ten foetal control brains ranging from 16 to 36 WG were selected. Main clinical and morphological characteristics are presented in Table 1. In 6 out of the 10 cases, a medical termination of pregnancy (TOP) was carried out for pathologies other than cerebral. Two out of the 10 cases were in utero foetal death (IUFD), 2 cases were *perpartum* or immediate *postpartum* death with a cause other than cerebral or with no found cause after careful examination of the placenta and foetal organs. In all cases, the brain was macroscopically and microscopically free of detectable abnormalities. Despite the absence of lesions, patients who had been antenatally suspected of central nervous system anomalies or who had been clinically suspected of dying of neurological causes were systematically excluded.

**Table 1:** Gestational age and cause of death of selected control cases

Case number	Term	Cerebral maturation*	TOP	Cause of death
1	16 WG	16 WG	Yes	Isolated sacral myelomeningocele
2	22 WG	20 WG	No	IUFD
3	22 WG	22 WG	Yes	Obstructive uropathy
4	24 WG	24 WG	No	IUFD
5	26 WG	26 WG	Yes	Hereditary bilateral microphtalmia
6	28 WG	28 WG	Yes	Severe distal arthrogryposis
7	30 WG	30 WG	No	Cord prolapse
8	32 WG	32 WG	Yes	Complex cardiac malformation
9	34 WG	34 WG	Yes	Suspected vermis hypoplasia (not confirmed)
10	36 WG	36 WG	No	Dilated cardiomyopathy Dead at day 2

*IUFD* in utero fetal death, *TOP* medical termination of pregnancy, *WG* weeks of gestation

*According to the morphometric criteria of Guihard-Costa and Larroche [17]

Fourteen foetal FAS or FASD brains ranging from 15 to 37 WG whose clinical and morphological characteristics are presented in Table 2 were also studied. Causes of death were IUFD in 6 cases, TOP for foetal malformations in 7 cases and post-natal early death in the remaining case.

**Table 2:** Main clinical and morphological characteristics of antenatal alcohol exposed fetuses

Case number	WG	Cause of death	Body weight	Cranio-facial dysmorphism	Brain weight	Visceral anomalies	CNS anomalies	Maternal alcohol intake	Maternal co-morbidity
1	15	TOP	25th percentile	Indistinct philtrum Low set posteriorly rotated ears microretrognathism	50th percentile 15.8 g	Anterior coelosomia	No	Chronic alcohol intake*	Heroin methadone
2	20	TOP Maternal distress	10th percentile	Midface hypoplasia, short nose, flat face	25th percentile 39.34 g	No	No	Chronic alcohol intake and Binge drinking* (ND)	HIV, Hepatitis C multi-drug addiction
3	22	TOP Chiari malformation	< 3rd percentile IUGR	FAS craniofacial dysmorphism	< < 3rd percentile 45.65 g	Renal hydronephrosis	Microcephaly Arnold Chiari II Myelomeningocele	Chronic alcohol intake**	Psychotic disorder Valproate
4	22	TOP Maternal distress	3rd percentile IUGR	Indistinct philtrum Posteriorly rotated ears Microretrognathism	50th percentile 63.5 g	No	No	Chronic alcohol intake (262 g per day) **	Psychotic Disorder psychotrops Increased MGV
5	24	TOP Amnion band sequence	NA	FAS craniofacial dysmorphism	50th percentile 75 g	NA	Micropolygyria Migration anomalies Vermis hypoplasia cerebellar hemisphere necrosis	Chronic alcohol intake**	Cocaine
6	24	TOP Septal agenesis	50th percentile	FAS dysmorphism	50th percentile 100.3 g	No	Arhinencephaly	Chronic alcohol intake**	Cannabis addiction
7	26	Spontaneous abortion	50th percentile	Indistinct philtrum Posteriorly rotated ears Microretrognathism	50th percentile 133.6 g	Unilateral pelvic dilatation	No	Chronic alcohol intake**	ND
8	27	Preeclampsia death at D10	10th percentile	FAS craniofacial dysmorphism	3rd percentile 109 g	No	Microcephaly Massive cerebellar haemorraghe	Alcohol intake**	ND
9	29	IUFD cardiopathy	5th percentile	Anteversed nostrils and pointed nose Ear anomalies Indistinct philtrum Retrognathism	5th percentile 178 g	Tetralogy of fallot	No	Daily chronic alcohol intake* (ND)	Increased MGV and GGT
10	30	IUFD Abruptio placentae	10th percentile	No	50th percentile 211 g	No	Bilateral intraventricular haemorrhage	Daily chronic alcohol intake*	Cannabis addiction Treated hypothyroidism
11	31	IUFD Preeclampsia	3rd percentile IUGR	Ear anomalies Retrognathism	3rd percentile 197.05 g	No	No	Daily chronic alcohol intake* (ND)	Increased MGV and GGT
12	31	TOP Septal agenesis	50th percentile	FAS craniofacial dysmorphism	50th percentile 234 g	No	Septal agenesis	Chronic and Binge drinking*	Cannabis
13	33	IUFD Acute alcohol Intoxication (maternal distress)	50th percentile	No	50th percentile 348.15 g	Amniotic fluid inhalation	Diffuse astrogliosis	Chronic and Binge drinking* (4.98 g/L)	Multi-drug addiction Increased MGV and GGT First pregnancy: IUFD at 33 WG One child alive with FAS
14	37	IUFD	25th percentile	Indistinct philtrum Microretrognathism	< 3rd percentile 248 g	No	Neuron heterotopia Microcephaly	Chronic and Binge drinking**	Heroin addiction

*CNS* central nervous system, *GGT* gamma-glutamyl transferase, *IUFD* in utero fetal death, *IUGR* intra uterine growth retardation, *MGV* mean globular volume, *NA* not available, *PN* post-natal, *TOP* medical termination of the pregnancy, *WG* weeks of gestation

*Maternal self report

**Suspected

## Methods

Autopsies had been performed according to standardized protocols [20]. Developmental age was evaluated by means of organ weights [17], skeletal measurements and by the histological maturational stages of the different viscera.

### Neuropathological studies

Brain growth was evaluated according to the biometric data of Guihard-Costa and Larroche [16]. Macroscopic analysis of gyration was assessed by means of the atlas of Feess-Higgins and Larroche [14]. After fixation into a zinc-10% formalin buffer solution for at least one month, brain sections were obtained from all cortical areas and deep subcortical structures. Seven-micrometer paraffin embedded sections were stained using Haematoxylin–Eosin. The morphology of the different brain structures analysed was consistent with the foetal age.

### Immunohistochemistry

Immunohistochemical analyses of OPCs and pre-OLs were carried out on six-micrometer sections obtained from paraffin-embedded material according to standardized protocols using antisera directed against Olig2 (Rabbit polyclonal, 1/200; Clinisciences, Nanterre, France) and anti-PDGFR-α (Rabbit polyclonal, 1/100; Thermofisher Scientific F67403 Illkirch Cedex, France). Noteworthy, some markers of mature oligodendrocytes such as myelin basic protein (MBP), myelin oligodendrocyte glycoprotein (MOG), phospholipid protein (PLP), adenomatous polyposis coli complex (APC) and cyclic nucleotide phosphodiesterase (CNPase) could not be used as they work on frozen tissues.

Immunohistochemical procedures included a microwave pre-treatment protocol to aid antigen retrieval (pre-treatment CC1 kit, Ventana Medical Systems Inc, Tucson AZ). Incubations were performed for 32 min at room temperature using the Ventana Benchmark XT system. After incubation, slides were processed by means of the Ultraview Universal DAB detection kit (Ventana). Semi-quantitative analyses of the density in Olig2 and PDGFR-α positive cells in the GE as well as in the different layers of the frontal CP were evaluated and scored blindly by two neuropathologists (FM and AL), and together reanalyzed in case of discrepant results. Immunolabellings were scored as follows: 0: no cell labelled; +: less than 10% of cells labelled; ++: between 10 and 25% of cells labelled; +++: between 25 and 50% of cells labelled and ++++: more than 50% of the cells labelled.

### Confocal analyses

In order to identify more precisely the different Olig2-positive populations, double immunolabelling was performed using Olig2 and either PDGFR-α, GABA, MAP2 or GFAP within the GE at 15–16 WG, a developmental stage in which some Olig2 positive cells could belong to other lineages than OL. The antibodies used for confocal analyses are described in Table 3. Brain sections were incubated overnight at 4 °C with various primary antibodies diluted in a buffer solution (PBS containing 1% BSA and 3% Triton X-100). Fluorescent-conjugated antibodies Alexafluor-488 and -592 were obtained from Molecular Probes (Eurogene, Or, USA). Sections were then rinsed three times with PBS for 10 min and incubated with the same incubation buffer containing the appropriate secondary antibody. Coverslips were mounted in DAPI-containing Vectashield (Vector laboratories, Cambridgeshire, UK). Non-specific binding of the secondary antibody was evaluated by omitting the primary antibodies. Images were acquired with the Leica laser scanning confocal microscope TCS SP2 AOBS (Leica Microsystems, Wetzlar, Germany). Analyses were carried out using the FIJI Is Just Image J (FIJI) software.

**Table 3:** Details of antibodies used for confocal analysis

Antibodies	Reference	Purified species	Supplier	Dilution	Target	Solution of incubation
GABA	A2052	Rabbit	Sigma-Aldrich	1/400	Neurotransmitter	1% BSA, 3% Triton X-100 in PBS
GFAP	Ab10062	Mouse	Abcam	1/200	Intermediate filament protein	1% BSA, 3% Triton X-100 in PBS
MAP2	M4403	Mouse	Sigma Aldrich	1/100	Brain microtubule-associated protein	1% BSA, 3% Triton X-100 in PBS
Olig2	AF2418	Rabbit	R&D System	1/200	Oligodendrocyte lineage transcription factor 2	1% BSA, 3% Triton X-100 in PBS
PDGFRα	AF1062	Goat	R&D System	1/200	Platelet derived growth factor subunit α	1% BSA, 3% Triton X-100 in PBS

## Results

### FAS Patient’s clinical and morphological characteristics

Among the 14 cases exposed to alcohol, 3 (21%) had intra uterine growth retardation (IUGR). All but 2 cases (86%) had cranio-facial dysmorphism which was characteristic of FAS in half of these cases, associating short palpebral fissures, smooth philtrum and thin vermillion border [40]. Five cases (36%) had microcephaly with a brain weight ≤ 3rd percentile. Nine cases (57%) had other CNS anomalies already described in FAS and FASD patients, such as myelomeningocele, arhinencephaly, polymicrogyria, neuronal heterotopias and cerebellar anomalies (see Table 2). Clastic lesions were observed in 5 cases (36%) and five other cases had associated visceral anomalies (anterior coelosomia, hydronephrosis, unilateral pelvic dilatation and tetralogy of Fallot), which are known to occur in case of PAE.

Daily chronic alcohol intake throughout the pregnancy was self-reported by 7 mothers/ 14 (50%). Three out of the 7 mothers also consumed episodic high doses of alcohol named «binge drinking». In the other 7 cases, maternal alcohol intake was suspected on the basis of reports by their relatives (family and/or friends) or clinically suspected at the time of foetal autopsy according to the criteria established by Riley et al., i.e., craniofacial dysmorphism characteristic of FAS (6/7 suspected cases), microcephaly (3/7 suspected cases), other CNS lesions (5/7 suspected cases) and IUGR (2/7 suspected cases) [40]. Ten mothers (71%) had co-morbidities notably multidrug addiction, antiepileptic drugs and psychotic traits.

### Semi-quantitative analysis of PDGFR-α and Olig2 immunohistochemistry in the cortical plate and ganglionic eminences

**Fig. 1:**
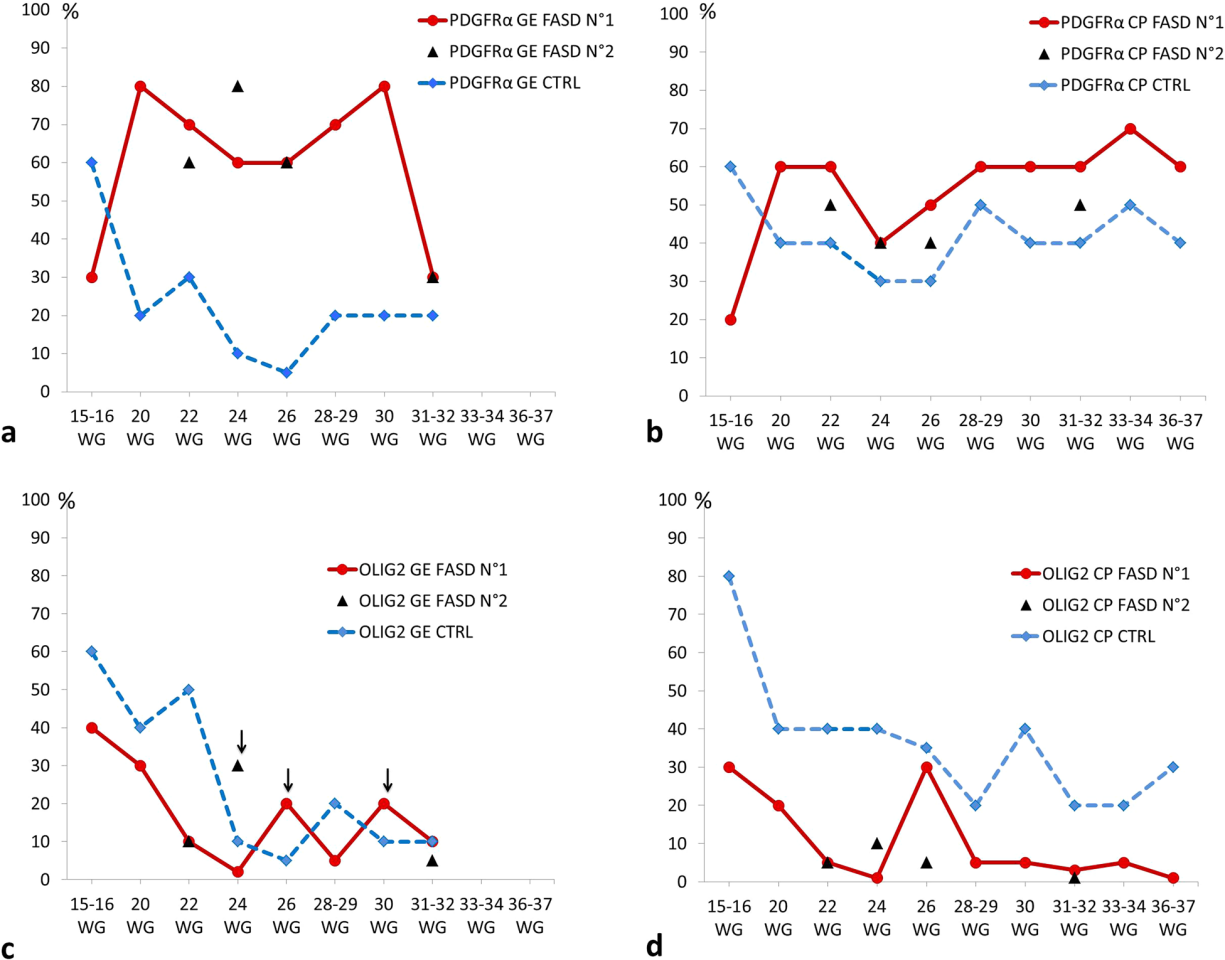
Schematic representation of PDGFR-α and Olig2 expressing cells in the GE and CP of FASD and control brains. Semi-quantitative evaluation of PDGFR-α immunoreactive OPCs in the GE displaying a delayed production at 16 WG and an increased density until the physiological disappearance of GE by comparison with control brains (**a**), as well as in the cortical plate of all FASD brains compared to control brains from 20 WG which persisted until 37 WG (**b**). Semi-quantitative evaluation of Olig2-expressing OPCs and pre-OLs in the GE in which the density of Olig2 immunoreactive cells was drastically reduced up to 24 WG in FASD brains, followed by an increasing trend to the production/differentiation between 24 and 30 WG (arrows) in FASD brains though the number of OPCs and pre-OLs remained low until regression of GE by 34 WG by comparison with control brains (**c**). In the cortical plate a lower density of Olig2-expressing cells in all FASD brains was observed regardless of the developmental stage compared with control brains (**d**). Dotted blue line: control brains; dotted red line: PAE exposed brains; black triangles: second FASD case available at a given stage

Semi-quantitative evaluation of PDGFR-α and Olig2 immunolabellings are summarised in Fig. 1 and in Table 4.

**Table 4:** Semi-quantitative analysis of immunohistochemical data with PDGFR-ɑ and Olig2 antibodies

Term	FASD/Ctrl	GE		CP	
		PDGFR-ɑ	Olig2	PDGFR-ɑ	Olig2
15 WG	FASD	30%+++	40%+++	20%++	30%+++
16 WG	CTRL	60%++++	60%++++	> 60%++++	80%++++
20 WG	FASD	80%++++	30%+++	60%++++	20%++
	CTRL	20%++	40%+++	40%+++	40%+++
22 WG	FASD 1	70%++++	10%+	60%++++	5%+
	FASD 2	60%++++	10%+	50%+++	5%+
	CTRL	30%+++	50%++++	40%+++	40%+++
24 WG	FASD 1	60%++++	2%+	40%+++	1%+
	FASD 2	80%++++	30%++	40%+++	10%+
	CTRL	10%+	10%+	30%+++	40%+++
26 WG	FASD 1	60%++++	20%++	50%+++	30%+++
	FASD 2	60%++++	NA	40%+++	5%+
	CTRL	5%+	5%+	30%+++	35%+++
29 WG	FASD	70%++++	5%+	60%++++	5%+
28 WG	CTRL	20%++	20%++	50%+++	20%++
30 WG	FASD	80%++++	20%++	60%++++	5%+
	CTRL	20%++	10%+	40%+++	40%+++
31 WG	FASD 1	30%+++	10%+	60%++++	3%+
	FASD 2	30%+++	5%+	50%+++	1%+
32 WG	CTRL	20%++	10%+	40%+++	20%++
33 WG	FASD	Absent	Absent	> 60%++++	5%+
34 WG	CTRL	Absent	Absent	50%+++	20%++
37 WG	FASD	Absent	Absent	60%++++	1%+
36 WG	CTRL	Absent	Absent	40%+++	30%+++

*CP* cortical plate, *CTRL* control, *FASD* fetal alcohol spectrum disorder, *GE* ganglionic eminences, *NA* not analyzed

**Fig. 2:**
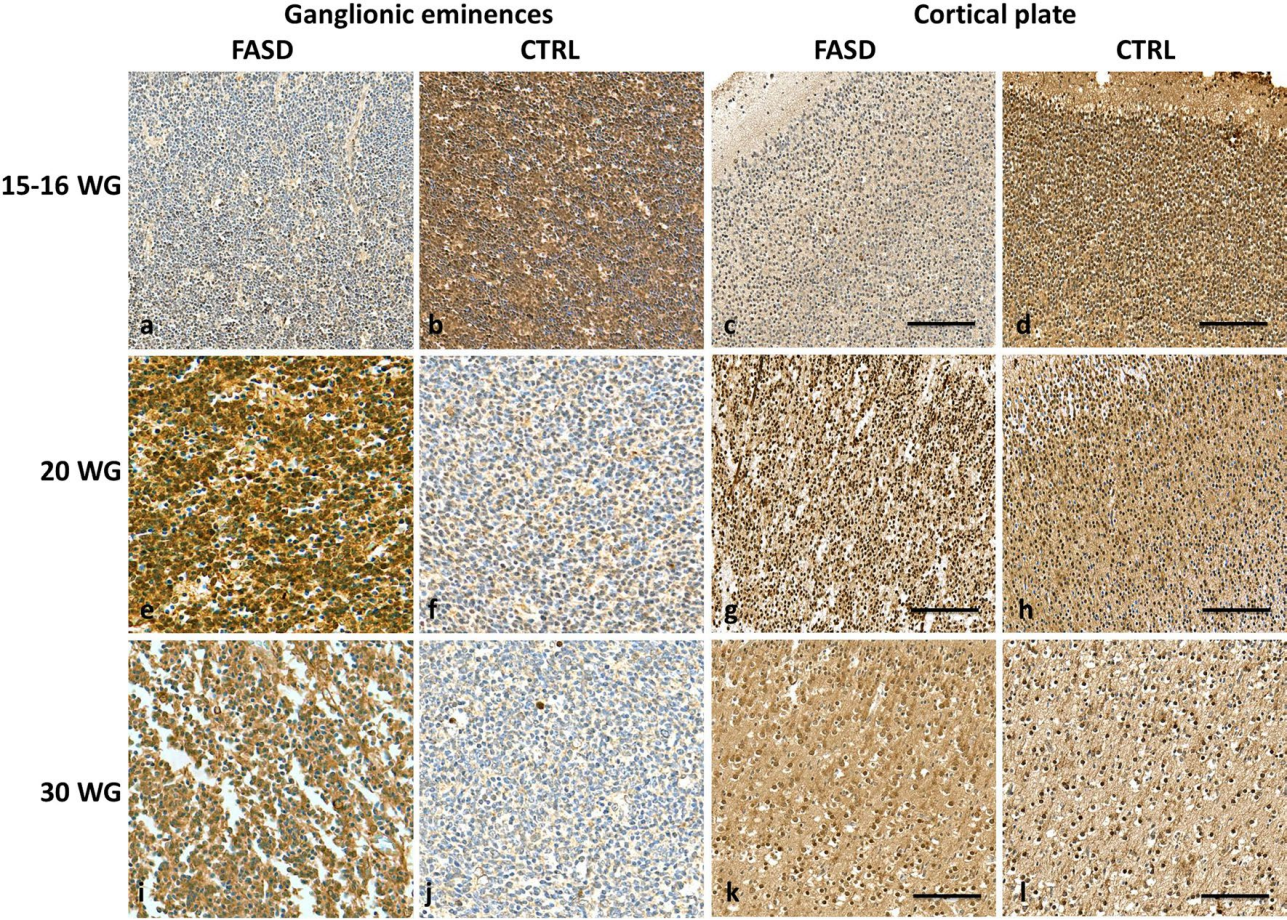
PDGFR-ɑ immunoreactivities in the GE and CP of FASD and control brains. Lower densities of PDGFR-ɑ expressing OPCs in the GE of FASD brain at 15 WG by comparison with the control aged 16 WG in which most of the cells were immunolabelled (OM X20) (**a, b**), with a similar pattern observed in the CP of FASD and control brain (**c, d**). But from 20 WG, higher densities of PDGFR-ɑ expressing OPCs in the GE of FASD brains compared with control brains (OM X20) (**e, f**), as observed in the CP (**g, h**), with the same pattern found at 30 WG in the GE (OM X20) (**i, j**) and in the CP (**k, l**). (OM: original magnification; scale bar: 0.35 mm)

PDGFR-α expression was strongly increased in the GE and in the CP of all FASD brains in comparison with controls whatever the developmental stage with the exception of the earliest stage (Fig. 1a, b). Indeed, at 15–16 WG, PDGFR-α expression remained lower in the medial ganglionic eminences (MGE), LGE and CP of FASD brains contrary to controls (Fig. 2a–d) in which more than 60% of cells were observed. At 20 WG and at all later stages, PDGFR-α cell numbers were higher than in controls in all structures studied (Fig. 2e–l).

**Fig. 3:**
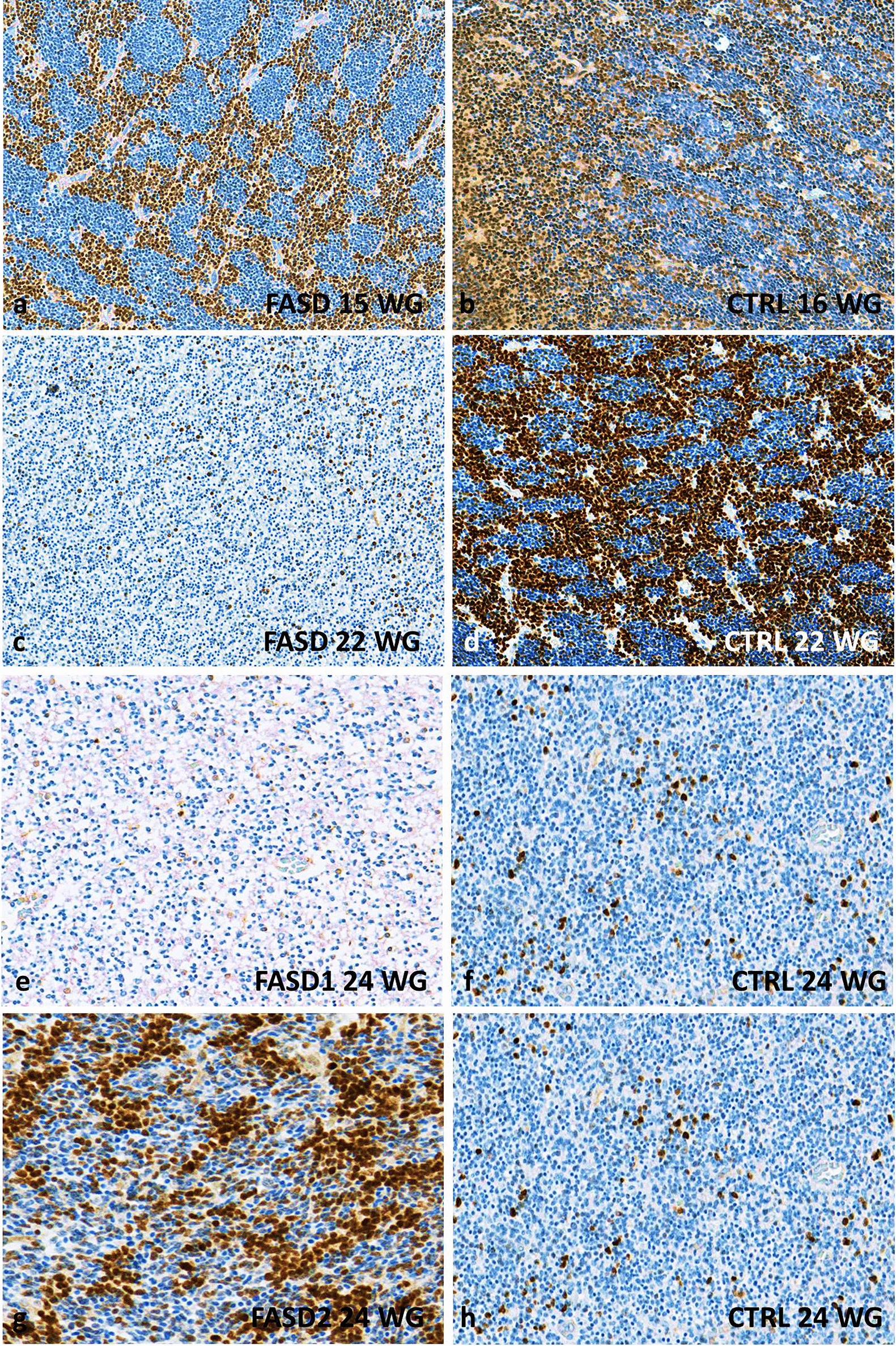
Olig2 immunoreactivities in the GE of FASD and control brains. Lower densities of Olig2 expressing cells in the GE of FASD brain at 15 WG by comparison with the GE of control brain at 16 WG in which more than 50% of Olig2 positive cells were observed (**a, b**) the most striking differences between FASD and control GE being observed at 22 WG (**c, d**). Similar differences, though less pronounced, were also noted at 24 WG in the FASD brain (case 5) compared to the control (**e, f**) contrary to what was noted in the FASD brain (case 6), in which an intense immunoreactivity was observed arguing for a delayed production/differentiation starting from this term (**g, h**)

At 16 WG, more than 60% Olig2-positive precursors and pre-OLs were identified in the MGE and LGE of the control brain, whereas Olig2-positive cells were less numerous in the FASD brain (Fig. 3a, b). Moreover, Olig2 immunoreactivity was essentially identified in the MGE of the FASD brain and not yet in the LGE. At 22 WG, Olig2-positive cell density was drastically reduced in the GE of FASD brains (Fig. 3c–f), and remained low until the regression of GE that normally occurs around 34 WG. No massive generation of Olig2 immunoreactive cells was identified in FASD brains until the end of the pregnancy, except for three small peaks of generation in FASD brains between 24 and 30 WG (Figs. 1c, 3g, h), contrary to controls in which a major production was observed between 16 and 24 WG.

**Fig. 4:**
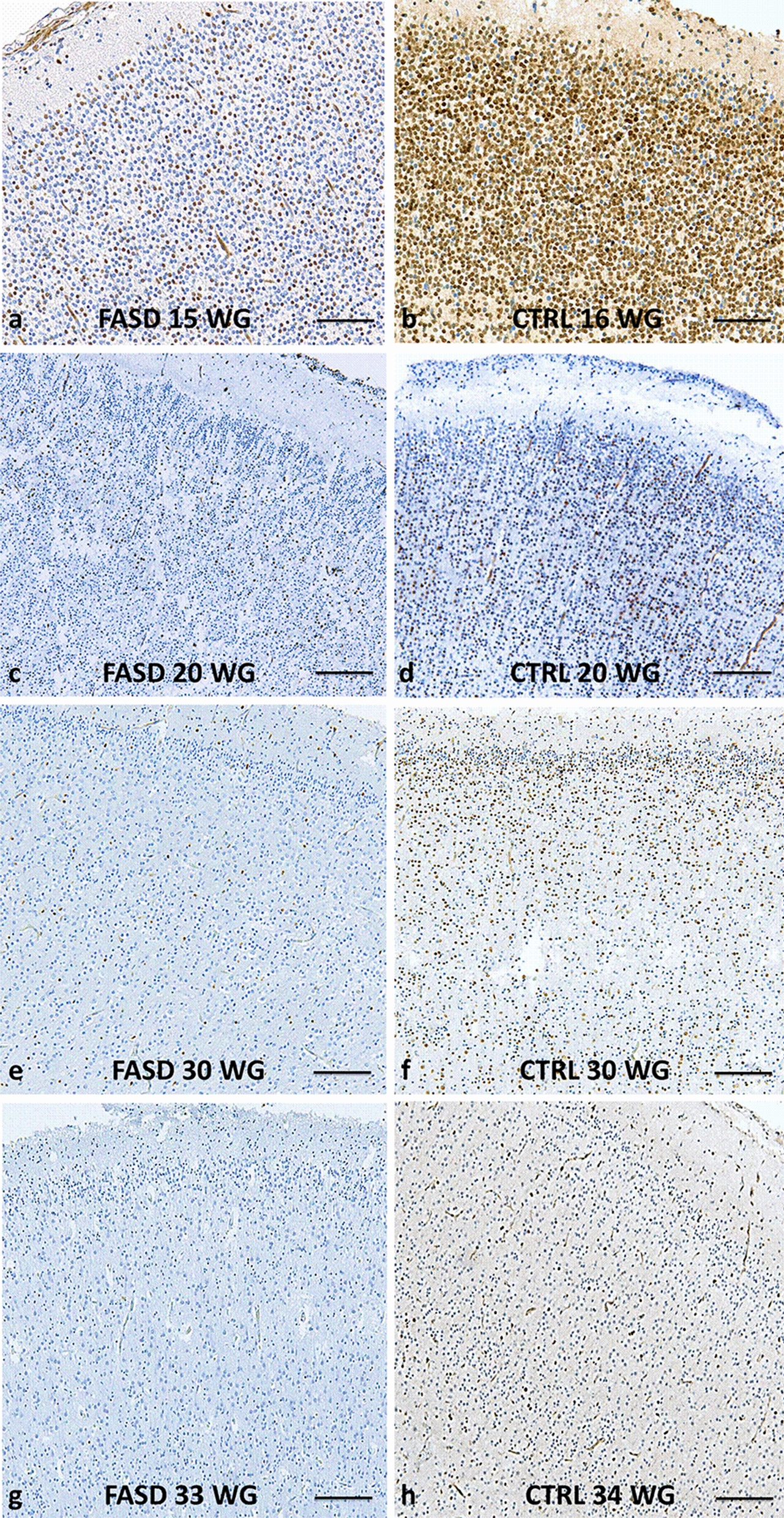
Olig2 immunoreactivities in the CP of FASD and control brains. Significantly lower densities of Olig2-positive cells in FASD brains compared to control brains at 15–16 WG (OM X10) (**a, b**) with a similar pattern observed at 20 WG (OM X10) (**c, d**). At 30 WG, Olig2-positive cells remained scarce in the FASD brain contrary to the control, in which Olig2-positive cells were located in all layers of the cortical plate (OM X20) (**e, f**) with an increase in density in the superficial layers at 33 WG in the control cortical plate only (OM X20) (**g, h**). (OM: original magnification; scale bar: 0.35 mm)

At all developmental stages, the density of Olig2-positive cells within the CP was consistently higher in each control brain in comparison with FASD brains (Figs. 1d, 4), with the highest density in the control cortical plate (around 80% of the cells) observed at 16 WG, contrasting with the paucity of immunolabelled cells in FASD (Fig. 4a, b).

### Confocal analyses

At early stages, a diffuse and intense immunolabelling of cells with Olig2 antibody was identified. As Olig2 is known to be also expressed in immature neurons and interneurons as well as in a subpopulation of astrocytes, we performed double immunolabellings using Olig2 and either MAP2, GABA, GFAP or PDGFR-α to evaluate more precisely the percentage of Olig2-positive OPCs [22, 23, 30]. No co-expression of Olig2 with MAP2, GABA, GFAP or PDGFR-α within the GE and CP was observed (Additional file 1: Fig. S1).

## Discussion

In living children with FASD aged from 10 to 17, imaging studies have revealed lower white matter volumes and sometimes a complete lack of myelination of major white matter tracts in the brain as well as white matter microstructure alterations [39, 48, 49]. However, the impact of alcohol on oligodendrocyte lineage and by extension on myelination has essentially been studied using in vitro experiments or animal models. Alcohol can disrupt myelination at any stage of OL development but targets particularly OPCs which are more vulnerable to excitotoxic damage, free radicals and pro-inflammatory cytokines than mature OLs [2, 39]. Furthermore, it has been shown in a study performed on primary mouse OL cultures that acetaldehyde, the metabolic byproduct of alcohol is lethal to OLs which are much more sensitive to acetaldehyde than to alcohol, particularly upon long-term alcohol exposure [6].

To our knowledge, no study concerning the effects of alcohol during human brain development has focused on OPC production from the GE. Nevertheless, using in vivo and in vitro mouse models, it has been demonstrated that alcohol hinders basal progenitor proliferation in the ventricular zone/subventricular zone (VZ/SVZ) by interfering with the cell cycle at G1-S transition from early development [38]. It might therefore be suggested that a similar mechanism occurs in the LGE and CGE, which could explain defective or delayed production of OPCs during foetal life. Finally, only one study by Darbinian et al. has been published concerning oligodendroglial lineage generation and differentiation in FAS human brains over the period covering the second trimester of pregnancy, from 12.2 to 21.4 WG [9]. By means of mRNA and flow cytometry analyses, these authors showed that ethanol (EtOH) exposure was associated with an increased proportion of cells that express protein markers for early OL progenitors and with a reduced proportion of cells expressing mature OL markers. Nevertheless, the use of brain homogenates in this study did not make it possible to determine in which specific regions oligodendrocyte production was affected, i.e., CP and/or GE. The present study shows not only that oligodendrocyte production is delayed in the GE but also that the proportion of cells expressing maturing OL markers is reduced in FAS brains later in development, supporting the hypothesis that this defect in differentiation persists at least until birth. Such alterations could be partly explained by an enhanced apoptosis as caspase-3 activation has been shown to be substantially increased in EtOH exposed human foetuses [9]. During rodent brain development, alcohol has also been shown to impair astrocyte and oligodendrocyte differentiation and to increase apoptosis [51]. Furthermore, a study performed in foetal macaque brains exposed to alcohol has shown that the decrease in OLs observed in comparison with control brains was linked to massive apoptosis, a single in utero alcohol exposure triggering widespread acute apoptotic death of OLs throughout white matter regions at a rate higher than 12 times compared to the physiological OL apoptosis rate [7]. This study also highlighted the fact that OLs become sensitive to the apoptogenic effect of alcohol at the time they are beginning to generate myelin constituents in their cytoplasm, i.e., when they become positive for MBP and negative for PDGFR-α, a fact we could not confirm in our human cohort, as myelination starts from birth only [7]. From this study, it could be suggested that in addition to a defective and/or delayed generation of OPCs, apoptosis also likely contributes to the decrease of Olig2 positive cells observed in human FASD brains, which was also observed by Darbinian et al. [9]. Epigenetic mechanisms could play an additional role in cell fate specification of brain precursor cells as alcohol is known to induce oxidative stress that alters gene expression, in particular Shh, which promotes expansion and specification of multipotent progenitors into OPCs and immature OLs [12, 35]. Upon alcohol exposure, several other signalling pathways through which alcohol may directly disrupt OPC differentiation and survival have also been implicated, such as PDGFRα, Wnt3a and Wnt5a, which regulate OL cell fate specification, differentiation and proliferation [21, 27, 46]. The increase in PDGFR-α positive OPCs observed in our study could therefore be related to a deregulation of Wnt signalling, preventing OPCs from progressing towards OL differentiation.

Whereas changes in OL morphology, maturation, differentiation and survival have been reported in third trimester-equivalent preclinical models of FASD [7, 8, 52], very little is known about the deleterious effects of alcohol on OL lineage derived from distinct telencephalic germinal zones [15, 18]. In 2017, Newville et al. found a drastic decrease in the number of mature OLs and proliferating OPCs within the corpus callosum of alcohol-exposed mice at postnatal day 16, but neither mature OLs nor OPCs derived from the postnatal SVZ were numerically affected, indicating ontogenetic heterogeneity in susceptibility to alcohol [33]. Several studies performed in rodents have demonstrated that myelination is delayed upon PAE, consisting in a weak expression of MBP, reduced myelin thickness and myelin alterations at the ultrastructural level, which impair the formation of neuronal circuits and conduction of neuronal signals [18, 32, 36].

Another mechanism which regulates the formation of myelin around axons consists in interactions between OPCs which receive excitatory and inhibitory inputs mediated by glutamate and GABA, and developing axons. Recent studies have demonstrated that a significant proportion of grey matter myelin in the cortex forms on the axons of local inhibitory interneurons in both rodents and humans [29, 44]. During development, GABA likely acts as a local environmental cue to control myelination and thus influences the conduction velocity of action potentials in the CNS. Nevertheless, data remain controversial since endogenous GABA by interacting with GABA_A_ receptors has been shown either to increase or to decrease proliferation, apoptosis and consequently oligodendrocyte numbers, as well as shortening internode length, which allows for faster saltatory conduction velocities. Similar discrepancies have also been observed regarding glutamate which blocks proliferation and progression of OPCs but also promotes myelin formation [19, 53].

It has long been acknowledged that in rodents, Olig2 expressing progenitors in the MGE give rise to GABAergic interneurons at early developmental stages and oligodendrocytes thereafter [30]. In humans, Olig2 has been mainly detected in the proliferative zones of the ganglionic eminences between 5–15 post-conceptional weeks prior to the expression of oligodendrocyte precursor markers. By 20 WG, these cells spread throughout the cortex, and co-express markers for immature neurons, neurogenic radial glia and intermediate progenitors [22, 23]. Using immunohistochemistry on 8–12 post-conceptional week human sections, Olig2 immunoreactivity has been shown to be expressed in GABAergic cells of the proliferative zones of the MGE and septum [1]. In the present study, the detection of more than 60% of Olig2 immunoreactive cells at the earliest stage could correspond to OPCs admixed with other nerve cell populations. Confocal studies together with quantitative analyses allowed us to show that Olig2 was specifically expressed in maturing OL from an early developmental stage and was not co-expressed with MAP2, GABA, GFAP or PDGFR-α.

## Conclusion

The present study provides further evidence that there is major oligodendrocyte lineage impairment at all stages of brain development upon PAE, consisting in defective/delayed generation, migration and maturation of oligodendrocyte precursors. Since oligodendrocyte development and myelin are a target of alcohol, the disruption of oligodendrocyte differentiation and of myelination process is very likely responsible for inadequate establishment of neuronal networks and inefficient conduction of neuronal signals. Disruption of oligodendrocyte generation and differentiation, together with GABA interneuronopathy that we previously identified [28], most likely contribute to developmental encephalopathy and subsequent life-long neuro-behavioural disabilities.

## Supplementary Information

**Additional file 1: Figure S1**. Confocal analyses in the GE of a normal brain at 16 WG Double immunolabellings using Olig2 (red) and MAP2 (green) (a), Olig2 (red) and GFAP (green) (b), Olig2 (red) and GABA (green) (c) and using Olig2 (red) and PDGFR-α (green) (d) did not reveal any co-expression with neurons, interneurons, astrocytes and oligodendroglial precursor markers

## Data Availability

Most data generated or analyzed during this study are included in this article. Additional datasets are available from the corresponding author on request.

## Abbreviations

CGE: Caudal Ganglionic Eminence
CNS: Central Nervous System
CP: Cortical Plate
DTI: Diffusion Tensor Imaging
FAS: Foetal Alcohol Syndrome
FASD: Foetal Alcohol Spectrum Disorder
GE: Ganglionic Eminences
IUFD: In Utero Foetal Death
IUGR: Intra Uterine Growth Retardation
LGE: Lateral Ganglionic Eminences
MBP: Myelin Basic Protein
MGE: Medial Ganglionic Eminence
OLs: Oligodendrocytes
Olig2: Oligodendrocyte lineage transcription factor 2
OPCs: Oligodendrocyte Precursor Cells
PAE: Prenatal Alcohol Exposure
PDGFRα: Platelet-Derived Growth Factor Receptor alpha
Pre-OL: Pre-Oligodendrocyte
Shh: Sonic Hedgehog
SVZ: Subventricular Zone
TOP: Termination of Pregnancy
VZ: Ventricular Zone
WG: Weeks Gestation
