# Supplementary figures and images for: Oligodendrocyte lineage is severely affected in human alcohol-exposed foetuses
Source: Acta Neuropathol Commun. 2022 May 14;10:74. doi: 10.1186/s40478-022-01378-9 (PMC9107108; doi:10.1186/s40478-022-01378-9)

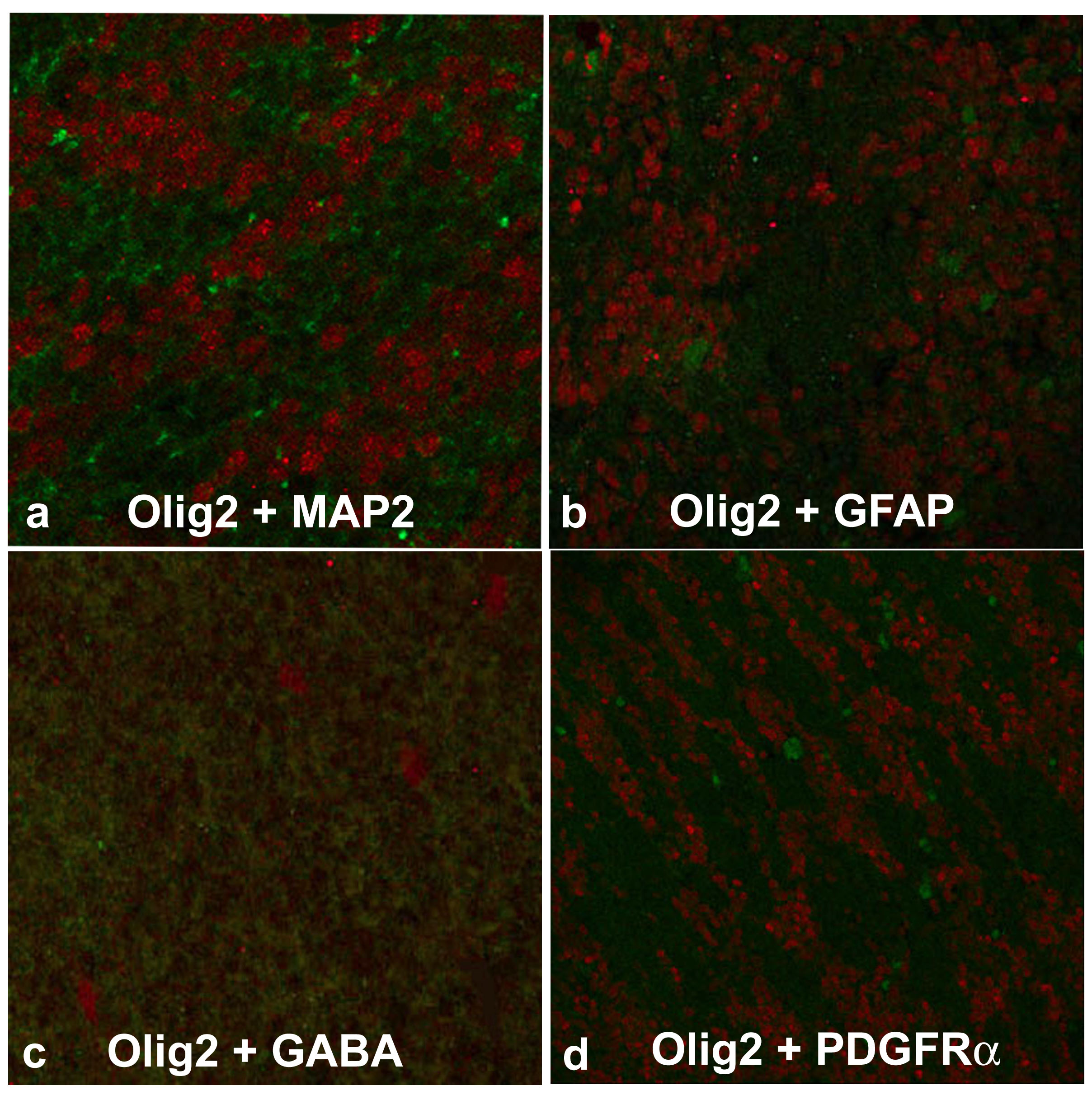

Supplement: Supplementary file 1 — Additional file 1: Figure S1. Confocal analyses in the GE of a normal brain at 16 WG Double immunolabellings using Olig2 (red) and MAP2 (green) (a), Olig2 (red) and GFAP (green) (b), Olig2 (red) and GABA (green) (c) and using Olig2 (red) and PDGFR-α (green) (d) did not reveal any co-expression with neurons, interneurons, astrocytes and oligodendroglial precursor markers [file 40478_2022_1378_MOESM1_ESM.jpg]
